# Simultaneous sulfate and nitrate reduction in coastal sediments

**DOI:** 10.1038/s43705-023-00222-y

**Published:** 2023-03-08

**Authors:** O. M. Bourceau, T. Ferdelman, G. Lavik, M. Mussmann, M. M. M. Kuypers, H. K. Marchant

**Affiliations:** 1 Max Planck Institute for Marine Microbiology, Celsiusstraße 1, 28359 Bremen, Germany; 2 University of Vienna, Department of Microbiology and Ecosystem Science, Division of Microbial Ecology, Djerassiplatz 1, A-1030 Vienna, Austria; 3 University of Bremen, Center for Marine Environmental Sciences, MARUM, 28359 Bremen, Germany

**Keywords:** Biogeochemistry, Biogeochemistry, Biogeochemistry

## Abstract

The oscillating redox conditions that characterize coastal sandy sediments foster microbial communities capable of respiring oxygen and nitrate simultaneously, thereby increasing the potential for organic matter remineralization, nitrogen (N)-loss and emissions of the greenhouse gas nitrous oxide. It is unknown to what extent these conditions also lead to overlaps between dissimilatory nitrate and sulfate respiration. Here, we show that sulfate and nitrate respiration co-occur in the surface sediments of an intertidal sand flat. Furthermore, we found strong correlations between dissimilatory nitrite reduction to ammonium (DNRA) and sulfate reduction rates. Until now, the nitrogen and sulfur cycles were assumed to be mainly linked in marine sediments by the activity of nitrate-reducing sulfide oxidisers. However, transcriptomic analyses revealed that the functional marker gene for DNRA (nrfA) was more associated with microorganisms known to reduce sulfate rather than oxidise sulfide. Our results suggest that when nitrate is supplied to the sediment community upon tidal inundation, part of the sulfate reducing community may switch respiratory strategy to DNRA. Therefore increases in sulfate reduction rate in-situ may result in enhanced DNRA and reduced denitrification rates. Intriguingly, the shift from denitrification to DNRA did not influence the amount of N_2_O produced by the denitrifying community. Our results imply that microorganisms classically considered as sulfate reducers control the potential for DNRA within coastal sediments when redox conditions oscillate and therefore retain ammonium that would otherwise be removed by denitrification, exacerbating eutrophication.

## Introduction

The permeable sandy sediments that fringe coastlines act as highly effective biocatalytic filters that remineralize organic carbon, and remove fixed nitrogen through denitrification [1–5]. The microbial communities that catalyze biogeochemical transformations in permeable sediments are subjected to frequent oscillations in electron acceptor supply, wherein the depth to which oxygen and nitrate penetrate the sediment can change in minutes [6–10]. These oscillations are due to changes in porewater advection resulting from changing tidal currents, waves, the shape of sandbed surfaces, and bio-turbation and bio-irrigation [4, 11, 12]. On longer time scales high currents and storm events mobilize sandy sediments, redistributing sand grains and their attached microorganisms between sediment layers [13–17].

Many of the microorganisms within permeable sediments appear to be adapted to the oscillating oxic and anoxic conditions [18]. Such adaptations include metabolic specialization of organisms involved in the process of denitrification, which leads to the removal of nitrate but also substantial nitrous oxide emissions [19, 20]. Furthermore, rapid shifts in redox conditions and electron acceptor availability result in microorganisms simultaneously using terminal oxidases and N-reductases. This leads to the co-occurrence of denitrification and aerobic respiration processes, which are typically spatially or temporally separated in diffusion limited sediments [10, 18, 19]. Potentially, sulfate reduction and nitrate reduction may also occur simultaneously in surface sediments where nitrate is intermittently supplied [21], or even sulfate reduction and oxygen respiration. However, the potential interactions between simultaneous sulfate reduction and pathways of nitrate reduction in permeable sediments remain unexplored.

Typically, microorganisms in marine sediments employ different electron acceptors over depth, largely in accordance with their decreasing energy yield, which often leads to an apparent spatial separation of sulfate reduction from nitrate reduction [22–24]. This separation is likely maintained by competitive exclusion, wherein N-reducers outcompete sulfate reducers because they conserve more energy per electron donated [24–27]. Furthermore, nitrite accumulation, which has been observed to occur due to metabolic specialization in sands [20], can also competitively inhibit sulfite reductase, an enzyme crucial for sulfate reduction [28, 29]. Nevertheless, sulfate reduction and denitrification can be linked via microbial activity [22]. For example, microbes can bridge the distance between sulfidic and nitrate-rich sediment by either migration [30] or electrogenic pili and perform sulfide oxidation coupled to nitrate reduction [31, 32]. When nitrate reduction is coupled to complete sulfide oxidation, sulfate reduction can therefore be underestimated [33–36].

Several lines of evidence suggest that sulfate reduction in intertidal permeable sediments should be tolerant of nitrate. Sulfate reducers are present and highly active in the upper layers of sediment, even though this area is frequently exposed to both nitrate and oxygen [6, 37–40]. A recent study has shown that sulfate reducing bacteria have higher acetate assimilation rates in the uppermost sediment layer than in deeper sediment layers [41]. Furthermore, in chemostat enrichments of intertidal permeable sediments, sulfide produced from sulfate reducers fueled denitrifier and ammonifier populations [42, 43]. Together, these studies suggest that sulfate reducers in permeable intertidal sediments can coexist with denitrifying microorganisms and could be adapted to, rather than inhibited by, frequent exposure to nitrate and even oxygen.

The co-occurrence of nitrate and sulfate respiration has the potential to impact N-removal pathways. For example, the presence of sulfide has previously been predicted to lead to higher nitrous oxide emissions during denitrification [44], and might enhance emissions of this potent greenhouse gas from permeable sediments. The occurrence of sulfate reduction might also alter the balance between denitrification and dissimilatory reduction of nitrate/nitrite to ammonium (DNRA), a process which retains fixed N in coastal systems rather than removing it. For example, the oxidation of sulfide produced by sulfate reduction has recently been linked to the DNRA community rather than the denitrifying community in coastal salt marsh sediments [45]. The link between sulfate and nitrate respiration could also be more direct, as many organisms that are traditionally thought of as sulfate reducing bacteria also have the potential to reduce nitrite to ammonium. Of these, some have been shown to switch to canonical DNRA when nitrate becomes available and use the pathway to support growth [46, 47], while others continue to preferentially reduce sulfate in the presence of oxidized N-compounds [48–50]. The reduction of nitrite to ammonia can be also catalyzed by sulphite reductase itself, although this conversion likely has no physiological benefit [28, 29]. Organisms such as *Desulfovibrio vulgaris* can prevent this competitive inhibition of sulphite reductase via constitutive expression of periplasmic cytochrome c nitrite reductase (Nrf) to remove the nitrite, although there are contrasting reports as to whether this is also linked to energy generation [51, 52].

In this study we hypothesised that the dynamic conditions typical for intertidal permeable sediments lead to simultaneous nitrate and sulfate respiration, analogous to previous observations of simultaneous aerobic and anaerobic respiration [18]. Furthermore, we investigated whether the co-occurrence of nitrate and sulfate respiration impacts the balance of denitrification, DNRA and N_2_O production and thereby the functioning of sands as biocatalytic filters. To test this, nitrate and sulfate reduction rates were determined simultaneously in freshly collected sediments from the upper two cm of the Janssand intertidal sand flat in the North Sea. Subsequently, flow-through cores comprised of the same sediments were used to gain mechanistic insights into how oscillations in NO_3_^-^ availability typically caused by tidal currents or storm events impact the balance of denitrification, DNRA, N_2_O production and sulfate reduction. We found strong correlations between DNRA and sulfate reduction rates, indicating a close link between the two cycles. To gain further insights into the potential metabolism of the microorganisms responsible for this link, we examined the phylogenetic affiliations of transcripts associated with nrfA, the key marker gene for DNRA.

## Results and discussion

### Sediment conditioning

Spatial and temporal overlaps between denitrification, DNRA, and sulfate reduction in permeable coastal sediments were investigated using both fresh surface sediment and surface sediment conditioned over five days to different electron acceptor supply. Sediment was conditioned immediately after collection in October 2018 using different nitrate regimes designed to mimic the variability that occurs in different sediment horizons *in situ*.

**Fig. 1:**
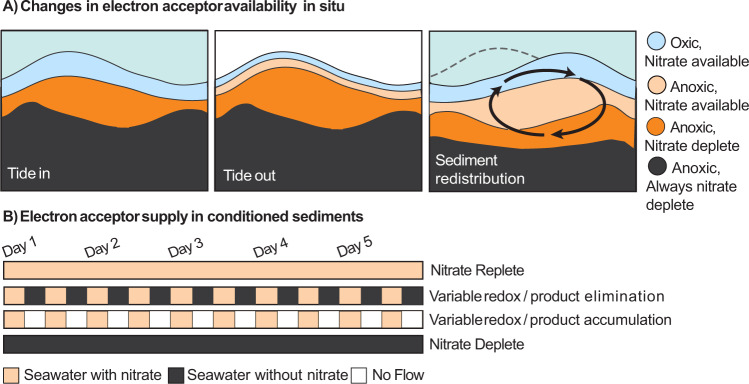
Exposure of the microbial community to variable NO_3_^-^ conditions in intertidal sand flats. **A** Changes in electron availability in situ. Schematic of the changes that occur on hourly to daily time scales on intertidal sand flats. When the tide is in, advection can transport O_2_ and NO_3_^-^ to depths of up to 5–10 cm. When the tide goes out, both are rapidly consumed and are only present in the upper mm to cm. When bottom currents become strong enough, or when wave action is high, the rippled sediment structures start to migrate, redistributing sand and exposing deeper sediments which have been NO_3_^-^ deplete for longer time periods. **B** Electron acceptor supply in the conditioned sediments: In addition to carrying out rate measurements on freshly collected sediments, sediments were exposed to different conditions over five days in flow-through reactors supplied with anoxic water.

On the tidal flat, oxygen and nitrate can penetrate to depths of 5–10 cm at high tide, but are quickly consumed when the tide recedes, whereupon they are only present in the upper mm [6, 53]. On longer time scales, sediment redistribution can bury the microorganisms that are attached to sand grains deeper into the sand flat, or, bring sand grains from deeper, more stably anoxic depths to the surface (Fig. 1A) [13, 14]. To mimic this variability in electron acceptor availability, two flow-through sediment cores were supplied with nitrate for 6 h, followed by a period of 6 h with no nitrate, similar to the upper layer of the sand flat (Fig. 1A). In one of these cores, flow was maintained constantly in order to remove metabolic products such as sulfide and Fe II (Variable Redox / Product Elimination), while in the other core, flow was stopped and metabolic products could accumulate (Variable Redox / Product Accumulation) (Fig. 1B). To mimic the conditions in the uppermost and deeper layers of the sediment, a third core was constantly supplied with nitrate-rich seawater (Nitrate Replete), while a fourth was constantly supplied with nitrate free seawater (Nitrate Deplete). All cores were kept anoxic throughout the conditioning period to isolate the effect of nitrate variations from those caused by oxygen.

During the five day conditioning period, the nitrate provided to the cores was consumed, indicating that nitrate reduction was likely occurring, or alternatively had been stored by the sediment diatom community [54]. Free sulfide was not detected in porewater at the outlet of any of the four cores, however substantial concentrations of dissolved Fe II were measured (Supplementary Fig. 2). Fe II release combined with rapid formation of black spots and gray sediment (indicative of iron sulfide formation) in cores receiving no nitrate (Supplementary Fig. 1), suggested the occurrence of substantial sulfate reduction in the cores [55].

At the end of the conditioning period, sediment from the center of the cores was sub-sampled in an anaerobic hood and 2 cm^3^ of sediment was placed into multiple 12 cm^3^ glass vials which were filled to the top with anoxic, filtered seawater before capping to create slurries. Rates of both sulfate reduction and nitrate consumption were then determined in the slurries in incubations amended with ^35^S sulfate tracer and no nitrate (Unamended incubations), or ^35^S sulfate and ^15^N-NO_3_^-^ (NO_3_^-^ Amended incubations). Nitrate reduction was determined in slurries receiving ^15^N-NO_3_^-^ but no ^35^S tracer. Throughout the incubation period the slurries were gently mixed by placing the glass vials in a roller tank to avoid the formation of nitrate-depleted microniches.

### Ubiquitous sulfate reduction

**Fig. 2:**
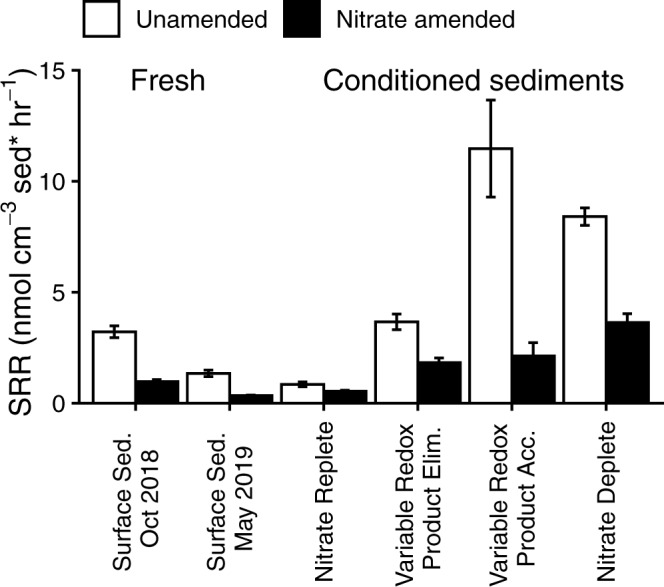
Sulfate reduction rates: Sulfate reduction rates (SRR) from sediments amended with NO_3_^-^ (black bars) or without NO_3_^-^ (white bars) in nmol cm^−3^ sed. hr^−1^. Sulfate reduction began immediately in all incubations, and rates are calculated from the period of time where rates were linear. See Supplementary Tables 1+2 for associated statistics. All rates had an adjusted R^2^ of at least 0.85, except for the Variable Redox / Product Accumulation condition, which had an R^2^ of 0.56 when amended with NO_3_^-^, and 0.75 without NO_3_^-^. All error bars represent standard error. The conditioned sediment incubations were carried out in October 2018.

Sulfate reduction occurred in all of the freshly collected and conditioned sediments when they were incubated without NO_3_^-^ (Unamended incubations) (Fig. 2, Supplementary Tables 1–3). Sulfate reduction rates in conditioned sediments (0.9–11.5 nmol cm^−3^ sed hr^−1^) varied substantially between cores, but overlapped with the range observed in freshly collected sediments (1.6–3.0 nmol cm^−3^ sed hr^−1^) and those measured previously in the upper two cm of the sand flat (0.42–16 nmol cm^−3^ h^−1^) [37, 56]. The rate of sulfate reduction in the sediment in the Variable Redox/ Product Elimination condition (1.8–3.7 nmol cm^−3^ sed hr^−1^) was most similar to that of the fresh sediment, indicating that this regime most closely simulated the surface sediments at the tidal flat. The occurrence of sulfate reduction in the surface layer of the sand flat, and in all of the conditioned sediments, including those that had been exposed to high NO_3_^-^ concentrations for five days (250–150 µM NO_3_^-^ in the variable cores) strongly indicates that sulfate reducers at the sandflat are acclimated to the recurring presence of NO_3_^-^ and sulfate reduction is therefore ubiquitous in these sediments. This is consistent with the observation of high acetate uptake by sulfate reducers in the surface sediment [41], and with the continued transcription of sulfate reduction genes in chemostats containing Janssand sediments after 100 days of continuous but low NO_3_^-^ exposure [42].

Nevertheless, the substantial differences in sulfate reduction rates between cores incubated with different NO_3_^-^ availabilities indicate that nitrate exposure does have some control over net sulfate reduction. Rates were 5 times higher in the Nitrate Deplete core (which had received no NO_3_^-^ for 5 days) than in the Nitrate Replete core (which had been constantly supplied with NO_3_^-^ for 5 days) (Fig. 2). Compared to the in situ rates, sulfate reduction rates were lower in the Nitrate Replete core, and vice versa, were higher in the Nitrate Deplete core. In contrast, in the Variable Redox conditioned sediments, NO_3_^-^ availability could not be clearly linked to the changes sulfate reduction rates. Although both Variable Redox sediments were exposed to similar nitrate regimes during the conditioning period, sulfate reduction rates were threefold higher in the flow-through core which had a stagnant period of 6 h (Variable Redox / Product Accumulation) in comparison to the reactor with constant flow (Variable Redox / Product Elimination).

### Co-occurrence of sulfate reduction with nitrate reduction

**Fig. 3:**
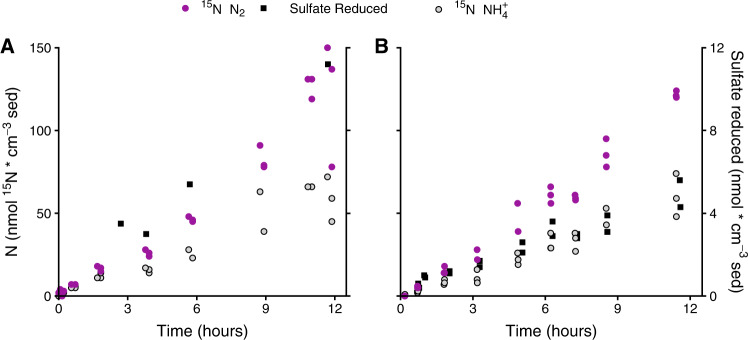
Concurrent NO_3_^-^ and sulfate reduction in freshly collected surface sediments: ^15^NH_4_^+^ (light gray circles) and ^15^N-N_2_ production (purples circles) and the total sulfate reduced (black squares) are plotted for parallel nitrate amended incubations of surface sediment collected in October 2018 (**A**) and May 2019 (**B**). Each point represents a measurement from a separate incubation. Note the different scale bars.

In the slurry incubations amended with 50–60 µM NO_3_^-^, sulfate and NO_3_^-^ reduction proceeded simultaneously in both the freshly collected and conditioned sediments (Fig. 3, Supplementary Fig. 5). In combination with the persistent sulfate reduction in NO_3_^-^ conditioned sediment (where no NO_3_^-^ was added during the incubation itself), these results suggest that the dynamic conditions at the sand flat select for a background level of constitutive sulfate reduction in anoxic permeable Janssand sediments, even in the presence a more thermodynamically favorable electron acceptor (NO_3_^-^). These results bear many similarities to the occurrence of denitrification in the presence of oxygen, previously observed in these sediments [10, 18].

Sulfate reduction always occurred in the presence of nitrate, albeit at ~20–60% of the rate observed in the unamended slurries (Fig. 2). There are at least three mechanisms that can explain this apparent decrease in sulfate reduction rates in the presence of NO_3_^-^; (1) competitive inhibition of sulfite reductase by NO_2_^-^ [29], (2) sulfate reducing bacteria switching their metabolism to DNRA [46, 47] and (3) complete sulfide oxidation to sulfate coupled to NO_3_^-^ reduction [57, 58]. Reported thresholds at which NO_2_^-^ completely inhibits sulfate reduction and/or growth by sulfate reducing bacteria vary greatly (0.04 mM – 10 mM), but are generally above the concentrations observed during our incubations, where NO_2_^-^ concentrations peaked at 35 µM (Supplementary Figs. 3, 4) [27, 59, 60]. While purified dissimilatory sulphite reductase has a high affinity (although low turnover) for NO_2_^-^ (K_m_ = 38 µM; k_cat_ = 0.038 mol s^−1^ mol^−1^ haem) [29] there was no obvious link between an accumulation of NO_2_^-^ and decreased sulfate reduction in the incubations (Supplementary Figs. 3–5). Therefore we suggest that the reoxidation of sulfide by sulfide oxidisers (sometimes referred to as cryptic sulfur cycling), or sulfate reducers switching their metabolism to DNRA are more likely explanations for the apparent decrease in sulfate reduction with NO_3_^-^ addition. However, it should be noted that sulfate reduction samples were processed using the Cr-II reduction method (Roy et al., 2014), which captures both produced sulfide and sulfur intermediate oxidation state compounds (e.g., pyrite, elemental S, thiosulfate, sulfite). As such re-oxidation of sulfide to sulfur intermediates would be included in the sulfate reduction rate determinations, but not any ^35^S-labeled sulfide that was rapidly and completely oxidized back to sulfate.

### Changing ratios of denitrification:DNRA in the presence of sulfate reduction

**Fig. 4:**
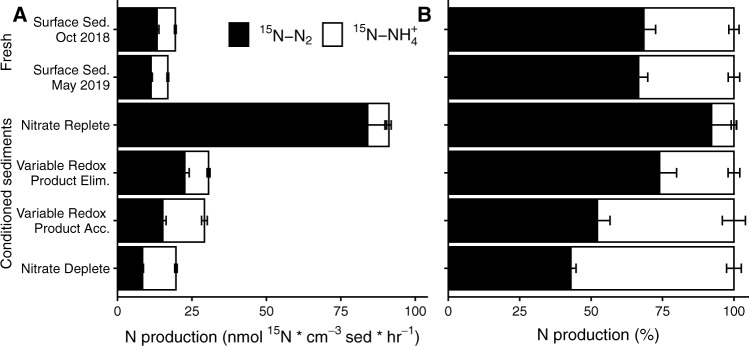
NO_3_^-^ reduction: **A** Rates of N_2_ (black) and NH_4_^+^ production (white) in nmol ^15^N cm^−3^ sed. in the four conditioned sediments and two freshly collected surface sediments. All error bars represent standard error of rates. All rates were fit to points where N_2_ production was approximately linear, and all rates have an R^2^ of at least 0.86 (see Supplementary Tables 1, 2 for associated statistic). **B** Normalized data from panel A showing the ^15^N-NO_3_^-^ converted to NH_4_^+^ or N_2_ as a percentage of the total rate of ^15^N-NO_3_^-^ conversion to either N_2_ or NH_4_^+^. Error bars represent propagated standard error. The conditioned sediment incubations were carried out in October 2018.

During the course of the incubations ^15^N-NO_3_^-^ was reduced to both ^15^N-N_2_ and ^15^N-NH_4_^+^, indicating that when nitrate was present there was the potential for both denitrification and DNRA to occur within the sediment (Fig. 4A). However the ratio of N_2_: NH_4_^+^ production differed substantially between sediments after they had been conditioned (Fig. 4B). For example, in the Nitrate Replete condition, denitrification was the dominant process and ^15^N- N_2_ production was 12 times higher than ^15^N- NH_4_^+^ production (Fig. 4). This was much higher than the ratio observed in the freshly collected surface sediments, where, as is typical for these sediments, denitrification was around twice as high as DNRA [54, 61]. Denitrification was also around twice as high as DNRA in the Variable Redox / Product Elimination sediment, while in the Variable Redox / Product Accumulation sediment, denitrification and DNRA rates were similar. Within the Nitrate Deplete core, DNRA was marginally higher than denitrification.

**Fig. 5:**
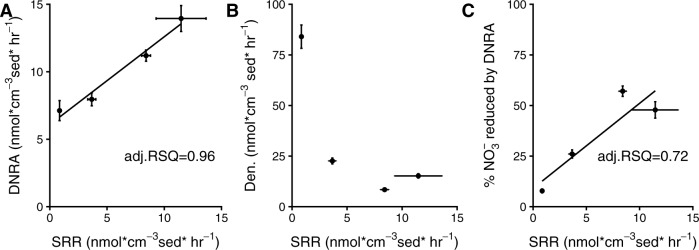
Correlations between sulfate reduction rates and nitrate reduction: **A** The rate of DNRA plotted against the sulfate reduction rate in the absence of nitrate. **B** The rate of denitrification (formation of ^15^N-N_2_) plotted against the sulfate reduction rate in the absence of nitrate. Vertical bars represent standard error and horizontal bars are the propagated standard errors of sulfate reduction rates. **C** The rate of ^15^NH_4_^+^ production (DNRA) as a percentage of the total production of reduced N (i.e. ^15^N-N_2_ + ^15^NH_4_^+^) plotted against the rate of sulfate reduction (SRR) that was determined in parallel incubations in the absence of nitrate. Horizontal bars represent standard error while vertical bars represent propagated standard error. These incubations were carried out in October 2018.

Different factors seem to have driven the changes in ratio in the different conditions, for example, in the Nitrate Replete condition, denitrification rates were far higher than those normally measured in the freshly collected sediments, while DNRA rates showed little change. This suggests that constantly anoxic, nitrate replete conditions allow the denitrification community to thrive in permeable sands. More interestingly, the relative contributions of DNRA and denitrification consistently varied with respect to sulfate reduction rates in the incubations (Fig. 5A, B), with the proportion of DNRA positively and strongly correlating with increased sulfate reduction rates (Fig. 5C). This suggests that sulfate reduction might exert an important influence on N-respiration when the processes co-occur.

### Linking microorganisms capable of DNRA to sulfur cycling

The correlation between denitrification to DNRA ratio and sulfate reduction was largely driven by increases in the DNRA rate (Fig. 5), rather than decreases in the denitrification rate (Fig. 5C), the latter of which was similar in the Variable and the Nitrate deplete conditions (Fig. 4B). In fact, DNRA rates in the Nitrate Deplete condition were more than double those measured in the freshly collected sediment, which suggests that the constantly anoxic, nitrate deplete conditions supported microorganisms capable of switching quickly to DNRA upon nitrate addition. Furthermore, the decrease in sulfate reduction rate that we observed upon addition of nitrate was also strongly correlated to the DNRA rate (Supplementary Fig. 6). These results suggest that DNRA could be linked to the re-oxidation of reduced compounds formed during sulfate reduction (i.e. Fe or H_2_S), or alternatively, that a portion of the sulfate reducing community may have switched to DNRA in the presence of nitrate. However, the complete re-oxidation of sulfide back to sulfate is notoriously hard to quantify experimentally in marine sediments [62], therefore we switched to an –omic approach to gain insights into the potential links between DNRA and sulfur cycling within these sediments.

**Fig. 6:**
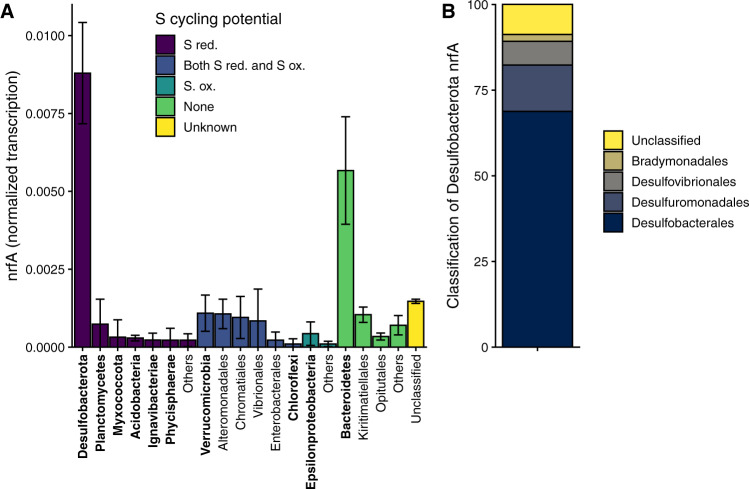
Transcription of nrfA, a key marker gene for DNRA in surface sediments at the sampling site **A** assignment of nrfA transcripts to phylum (bold), or order level. The colors indicate the potential of these classes to carry out sulfur metabolism as identified from literature surveys. Transcript abundance was normalized by gene length and against the total abundance of rpoB in the metatranscriptome. **B** Assignment of Desulfobacterota nrfA transcripts to order level, as a percentage of total nrfA transcripts assigned to Desulfobacterota. In both panels averages are shown from three individual metatranscriptomes and error bars are standard deviation. These samples were sequenced in 2015.

We examined the phylogenetic affiliations of nrfA transcripts (the key marker gene for DNRA) in three sediment layers at the sampling site (0–1 cm, 2–4 cm and 7–10 cm). On average, 90% of the identified nrfA transcripts could be taxonomically assigned to class level (Fig. 6). Transcript assignments were similar in all sediment layers, although relative levels of nrfA transcription were higher in the two deeper sediment layers (Supplementary Fig. 7). Around half of the transcripts were assigned to orders within the Desulfobacterota phylum (recently reclassified from the Deltaproteobacteria; see ref. [63]) which are associated with sulfate reduction; mainly Desulfobacterales, followed by Desulfuromonadales and Desulfovibrionales (Fig. 6B). In contrast, there were very few nrfA transcripts assigned to classes containing sulfide oxidizers, such as the Chromatiales and Woeseiaceae, which are common in these sediments [64, 65]. Most other nrfA transcripts were taxonomically assigned to a class that is rarely associated with dissimilatory sulfur metabolism; the Bacteroidetes and specifically, the families Bacteroidia and Flavobacteriia (Supplementary Fig. 7 and Supplementary Tables 4, 5), which are generally facultative anaerobes and fermenters.

The transcription of nrfA therefore suggests that DNRA in the sediment is largely carried out either by facultative anaerobes/fermenters and organisms that are classically considered to be sulfate reducers. Taken together our results indicate that the correlation between sulfate reduction and DNRA in the sediment is driven by sulfate reducing microorganisms switching between sulfate reduction and DNRA. This observation shows that the nitrogen and sulfur cycle in sediments can be linked by the direct activity of bacteria that switch electron acceptors, rather than, as is typically assumed, sulfide oxidation coupled to NO_3_^-^ reduction.

### Nitrous oxide production remained similar regardless of N-reduction pathway

**Fig. 7:**
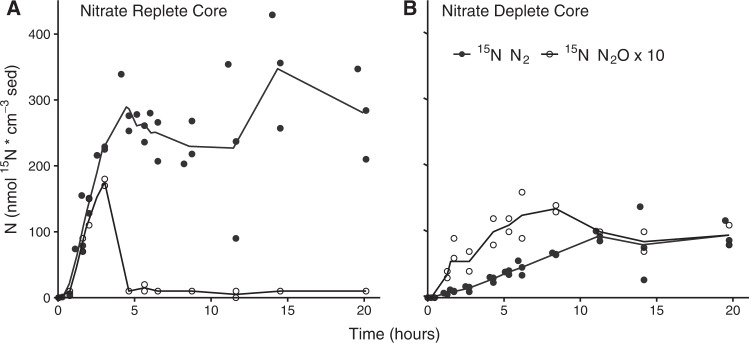
N_2_O and N_2_ production The production of N_2_ (black circles) and N_2_O (open circles, values multiplied by 10) in the Nitrate Replete core (**A**) and Nitrate Deplete core (**B**) over the entire incubation time in nmol ^15^N cm^−3^ sediment. Lines connect the average value at each timepoint. Each point represents a measurement from a separate incubation. These incubations were carried out in October 2018.

The accumulation of sulfide during S-cycling has also been suggested to impact N-cycling via the inhibition of nitrous oxide reductase, thereby decreasing N_2_ production and increasing N_2_O production [44, 66]. In contrast, there was only a weak negative correlation between sulfate reduction rate and N_2_ production rates in this study, and the correlation was mainly driven by the very high denitrification rate in the nitrate replete condition (Fig. 6). Changes in N_2_ production rate were not compensated for by large increases in N_2_O production, which represented only a few percent of total gaseous N production (i.e. N_2_O + N_2_) (Supplementary Table 6). Net N_2_O production occurred in all of the sediments in the first hours of the incubations, followed by net consumption as nitrate became limiting, as is typically observed in these sediments (Fig. 7, Supplementary Figs. 3, 4, 8, 9). Intriguingly, the net N_2_O production at the start of the incubations was similar regardless of the overall denitrification rate. This led to a substantial increase in the N_2_O:N_2_ production ratio at the start of the incubations in which denitrification rates were low and DNRA and sulfate reduction rates were high. Furthermore, there was a slower net reduction of N_2_O when NO_3_^-^ became limiting in these incubations. It is possible that the production of sulfide partially inhibited N_2_O reductase (although it should be noted that this would not have been a major driver of the denitrification:DNRA ratio). Alternatively, the production of Fe(II) in the incubations with higher sulfate reduction rates could have led to enhanced production of N_2_O by abiotic reactions [67]. Regardless of the mechanism, our results suggest that the release of the greenhouse gas N_2_O would not be reduced by a shift from denitrification to DNRA, despite the fact that DNRA itself does not release any N_2_O.

### Environmental implications of overlaps between sulfate reduction, DNRA and denitrification

Here we show that in coastal permeable sediments sulfate reduction occurs in nitrate replete sediments, where it overlaps with the processes of denitrification and DNRA, thereby increasing the volume of sediment in which sulfate reduction can occur. Nevertheless, sulfate reduction rates measured in freshly collected surface sediments were approximately 10–20% of the rate of N-reduction. This implies that while sulfate reducers seem to be tolerant to nitrate in the sediment, they only contribute to a minor proportion of total carbon turnover in the surface layer (0–2 cm), as has been noted for other permeable intertidal sediments [8].

**Fig. 8:**
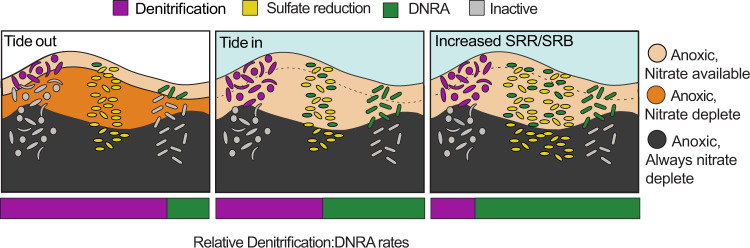
Influence of changing boundary conditions on process rates Schematic outlining the changes in microbial activity over a tidal cycle (left and middle panels) and in a case where sulfate reducing bacteria become more abundant (right panel). When the tide is out, only the upper surface of the sediment has nitrate, and nitrogen reduction is dominated by denitrification. When the tide is in, nitrate reaches deeper into the surface and correspondingly to more sulfate reducers, which switch their metabolism to DNRA. This results in a more even denitrification:DNRA ratio. In sediments with more sulfate reducers, it is expected that DNRA rates would also increase as some sulfate reducers perform DNRA. For simplicity, oxygen dynamics are neglected.

Furthermore, we found that a substantial proportion of DNRA in the sediment appears to be performed by organisms considered to be classical sulfate reducers. The ability of these microorganisms to respire and even grow via nitrate reduction has long been recognized and interestingly has also been associated with a high tolerance to oxygen exposure [48, 49, 68]. However, the reduction of nitrate as respiration strategy by sulfate reducers in marine sediments has rarely been observed; likely because sulfate reduction is generally considered to occur only in stable, nitrate deplete, anoxic environments. In contrast, organisms that would typically be classed as sulfate reducers, appear to be key members of the microbial community in permeable sediments where there are rapid fluctuations between fully oxic and nitrate replete conditions and anoxic and nitrate deplete conditions (Fig. 8). As a consequence, sulfate and nitrate reduction do not only co-occur in the sediment, but are directly linked within the *Desulforbacterota*. This implies that the size and activity of the sulfate reducing community controls the potential for DNRA within these sediments (Fig. 8). This could also explain the enhanced DNRA activity and increased ammonium fluxes to the water column that have been observed in sediments underlying hypoxic water columns [69]. This contrasts with the common view that the ratio of electron donor to nitrate/nitrite is the major factor driving the balance between DNRA and denitrification [70–72]. As DNRA retains fixed nitrogen within ecosystems as ammonium, rather than removing it like denitrification, our results indicate that the presence of active sulfate reducing communities can influence eutrophication.

## Methods

### Sampling site

Sediments were collected from the Janssand sand flat, a sandy intertidal area that lies in the back barrier region of the island of Spiekeroog, in the Wadden Sea, North West Germany i.e. between the island and the mainland, detailed site descriptions are available in [6, 56, 73]. The flat has a semidiurnal tidal cycle, whereby it is inundated with water for 5–6 h during high tide and exposed for 6–7 h during low tide [6, 73]. The upper flat has a mean grain size of 176 µm, a porosity of 0.35 and a permeability of approximately 7.2 * 10^−12^ m^2^ [6, 56]. When the sand flat is inundated with seawater, the interaction of bottom water currents with rippled sediment topography lead to variable advection of seawater into the sediment and O_2_ penetration depths vary between 1 and 5 cm [7, 10, 53]. During low tide O_2_ and NO_3_^-^ within the porewater are quickly depleted and O_2_ penetration depths drop to <1 cm [10, 54].

### Fresh sediment incubations

PVC core liners (I.D. 3.5 cm) were used to collect three vertical cores from the upper sand flat of Janssand during low tide on two occasions (October 17, 2018 and May 22, 2019) and transported to the lab (~2 h). In October surface water was approximately 14 °C, and in May 11 °C.

Cores were transferred to an anaerobic chamber and the upper pale (oxidized) layer (0–3 cm) was separated from a dark (reduced) layer (7–10 cm) (Supplementary Fig. 1). The upper layer was well mixed before 2 cm^3^ aliquots of sediment was transferred into 12 mL glass vials with septa (LabCo, Manchester), hereafter referred to as “Exetainers”, that were filled with filtered anoxic seawater collected October 10, 2018 (NO_3_^-^ + NO_2_^-^- < 2 µM) creating sediment slurries. Exetainers were capped headspace free and removed from the anaerobic chamber whereupon they were assigned to one of three treatment groups (Supplementary Fig. 1). 38 Exetainers per core received 60 µM ^15^N-labeled NO_3_^-^ (corresponding to ~300 nmol/cm^3^ sediment), 24 received 60 µM ^15^N-labeled NO_3_^-^ and 250 kBq of ^35^S-labeled sulfate, 24 received only 250 kBq of ^35^S-labeled sulfate. Filled Exetainers were placed in roller tanks on a roller table. The roller table speed was set in order to gently invert the Exetainers every 44 seconds along their longitudinal axis to ensure that the slurries remained homogenous. Visual observations confirmed that this constantly mixed the sediment with the seawater in the vials.

Slurries were weighed and killed in duplicates at 12 selected time points with the aim of including timepoints before and after NO_3_^-^ depletion. Slurries without added ^35^S (i.e. those with only ^15^N) were killed by injecting 100 µL 30% w/v zinc chloride and 200 µL saturated mercury chloride so that they were suitable for later ^15^N gas analysis. Slurries with added ^35^S were killed by first removing 1.8 mL sample water that was directly pipetted into 200 µL 20% w/v zinc acetate (total radioactivity samples) and stored at 4 °C, and the remaining sediment and water was decanted directly into 50 mL falcon tubes pre-filled with 7 mL 30% zinc acetate (TRIS samples) and frozen at −20 °C.

### Conditioned sediment incubations

Sediment from the upper 2 cm of the sand and approximately 70 L of surface seawater (~13 °C, NO_2_^-^ + NO_3_^-^- < 2 µM) was collected on October 10, 2018 during low tide and transported to the lab (~2 h). The seawater was filtered (polyethersulfone filters, .2 µm pore size) and stored in the dark at 4 °C for use in flow-through cores and incubations.

#### Core set up

The surface sand was homogenized and filled into four cylindrical acrylic cores with a 9 cm inner diameter following [5]. The radial groves surrounding a center port in the base of the cores was protected with 500 µm nylon mesh (Hydra-BIOS, Germany) to facilitate plug flow. Cores were filled gently with sand to 23–27 cm while immersed in freshly collected seawater to avoid trapping air bubbles. Filled cores were capped and then connected to a peristaltic pump at the base using viton tubing (Supplementary Fig. 1).

Cores were assigned to 1 of 4 conditions (Table 1) and filtered seawater previously deoxygenated by bubbling with N_2_ (and subsequently kept under a N_2_ headspace) was pumped into the bottom of the core according to the four conditioning regimes for five days. The core intended to mimic surface sediment, the Nitrate Replete condition, received a constant supply of NO_3_^-^-rich water, while the core intended to mimic deep sediment, the Nitrate Deplete condition received a constant supply of NO_3_^-^-poor seawater (Fig. 1). Two additional cores were intended to mimic portions of sediment with variable NO_3_^-^ availability. In the first, the Variable Redox / Product Elimination condition, water flow was constant, bringing NO_3_^-^ rich water for 6 h followed by NO_3_^-^ poor water for 6 h. In the second, the Variable Redox / Product Accumulation condition, NO_3_^-^ rich water was provided for 6 h and then the water was allowed to stagnate in the core for 6 h. Seawater for NO_3_^-^ amended cores was provided with 200 µM NO_3_^-^ for the first two days and thereafter 400 µM to ensure NO_3_^-^ availability throughout the testing zone (4–10 cm from the inlet; see Supplementary Table 7). Seawater was provided at a rate of ~50 mL per hour during pumping and therefore had a residence time of ~11 h in the cores with constant water flow (Nitrate Replete, Nitrate Deplete and Variable Redox / Product Elimination conditions). The influence and availability of O_2_ was minimal, as inlet water had been degassed by N_2_ bubbling. Sulfide and Fe II were determined using methylene blue [74, 75].

**Table 1:** Conditioning regimes.

Core	Nitrate amendment	Flow
Surface Sediment Proxy	+	Constant
Variable Redox + Product Build-up	+	6 h on, 6 h off
Variable Redox + Product Removal	+	6 h on
	–	6 h on
Deep Sediment Proxy	–	Constant

Flow through cores made up of the top 2 cm of the sand flat were conditioned with different water and nitrate regimes. Two cores had changing water provision and two had constant. The Variable Redox + Product Removal switched water sources every six hours, from one with nitrate to one without nitrate.

#### Conditioned sediment ^15^N & ^35^S incubations

After 5 days pre-conditioning, NO_3_^-^ and sulfate reduction rates were determined for sediment from each core. Each core was placed in an anaerobic chamber under an N_2_ atmosphere and sediment was sampled from 4 to 10 cm above the core base and homogenized. Sediment was then transferred to Exetainers (Labco, Manchester) to create slurries whereupon labeling with ^15^N-NO_3_^-^, ^35^S-sulfate and subsequent sampling was carried out identically to the fresh sediment incubations. ^35^S-sulfate labeled samples from T0-T2 in the Nitrate Replete and Nitrate Deplete conditions, and T0 in the variably conditioned sediments were not weighed before they were decanted into zinc acetate, therefore the average sediment mass from other samples in their respective treatments was used for rate calculations.

### Sulfur and nitrogen rate determinations

#### Determination of sulfate reduction rates

Sulfate reduction rates were determined according to Roy et al. [76]. Briefly, the zinc-preserved ^35^S samples were treated with a cold chromium acid distillation to extract the total reduced inorganic sulfur content (TRIS) containing ^35^S. Total ^35^S radioactivity in the supernatant, and TRIS ^35^S radioactivity was determined for each individual exetainer on a liquid scintillation counter (Tri-Carb 4910 TR Liquid Scintillation Analyzer, Perkin Elmer) using Ultima-Gold scintillation fluid (Perkin-Elmer). The total amount of sulfate reduced per sample was calculated with Eq. (1) adapted from [76]. Rates were determined by plotting the total sulfate reduced over time and applying linear fits. In the October fresh sediment incubations, an inconsistent amount of tracer was injected into the exetainers, so only those exetainers containing more than 20 kBq ^35^S at measurement were included.

1 $$SO_4^{2 - }reduced\left( {\frac{{{{{{{{{\mathrm{\mu }}}}}}}}mol}}{{cm^3}}} \right) = \frac{{Decays\;per\;minute_{TRIS}}}{{Decays\;per\;minute_{Total}}} \ast 1.04 \ast \left[ {SO_4^{2 - }} \right]$$

Sulfate concentrations $\left( {\left[ {SO_4^{2 - }} \right]} \right)$ in the exetainer incubations were determined by ion chromatography (Metrohm 9300 Compact IC Flex with in-line zinc-trapping column), and the average value (calculated without outliers from dilution error) for each time series was used for subsequent rate calculations.

#### Nitrogen measurements

A 2 mL helium headspace was created in the Exetainers (Labco, Manchester) to which ^15^N had been added. The liquid that was removed during this procedure was then used for NOx and ^15^NH_4_^+^ measurement.

#### NOx measurement

The NOx concentration of water was determined photometrically (Infinite M200 Pro, Tecan) using a version of the Griess reaction modified to sequentially determine NO_3_^-^ and NO_2_^-^ at low concentrations in small volumes [77, 78].

#### ^15^N- N_2_ measurement

^15^N*-*N_2_ concentrations were measured with a GC-IRMS (Isoprime PrecisION, Elementar). In total, 100 µL gas subsampled from the headspace of the Exetainers was injected directly into the GC-IRMS to determine the relative abundance of 29 and 30 N_2_. A standard curve of ambient air injections was then used to calculate gas concentrations according to [79]. Values were corrected for gas dissolved in water removed during headspacing. The sum of ^15^N-N_2_ production at each time point was calculated as (^29^N_2_ + (2 * ^30^N_2_)).

#### ^15^N N_2_O measurement

After ^15^N*-*N_2_ measurement, the Exetainers were spiked with 60 µL N_2_O and allowed to equilibrate overnight. Samples were measured as above, but injecting 250 µL gas, detecting ^45^N_2_O and ^46^N_2_O, and with an N_2_O standard curve. Values were corrected for N_2_O solubility and for gas dissolved in water removed during headspacing. In October N_2_O was only measured in two exetainers per time point, while in May three exetainers were measured per timepoint.

#### ^15^N-ammonium measurement

^15^N-ammonium production was determined after oxidation to N_2_ according to [80, 81]. ^15^N-N_2_ in the headspace was then measured as above. ^15^N- NH_4_^+^ standards were converted concurrently to ensure that conversion efficiency was always > 95%.

#### Rate calculation

Linear regression was performed on the data in order to calculate rates (Supplementary Table 1–3).

### Metatranscriptomics

Metatranscriptomics was performed on nine samples collected in July 2015 previously described in ref. [38]. Briefly, sediment was sampled at late low tide from the Janssand sand flat using three sediment cores. Cores were immediately sliced into 3 layers (0–1 cm, 2–4 cm and 6–8 cm) according to sediment color (brownish, brown to gray and gray to black), which is representative of the oxidized/sulfide free upper sediment zone, the sulfide transition zone and the reduced sulfidic zone. Sediment was transferred into 50 ml tubes within 20 s and immediately stored on dry ice or at −80 C until further processing. Total RNA was extracted after treatment with DNA-ase, purification and bacterial rRNA depletion before construction of RNA TrueSEQ libraries. These were sequenced paired end on an Illumina HiSeq platform (see ref. [38] for further details).

Transcripts of nrfA and rpoB were identified in the metatranscriptomes using the ROCker-approach detailed in Marchant et al. (2018). ROCker models were built according to ref. [82] using using a collection of curated protein sequences and in the case of nrfA, closely related outgroup protein sequences (downloaded from http://enve-omics.ce.gatech.edu/rocker/models). For comparison between metatranscriptomes, nrfA transcript read numbers were normalized against rpoB transcript read numbers and the corresponding size of each gene before calculating an average for each of the three replicate sediment layers. The taxonomic identity of the transcripts was inferred using Kaiju [83] (Genbank nr_euk database downloaded on 04. Aug. 2020) and samples were grouped to at least class level where possible. The capacity of organisms contained within each class to utilize S-compounds as either an electron donor or acceptor was then assigned based on literature searches (see in particular refs. [84] and [85]). For the Deltaproteobacteria and Bacteroidetes (which were the classes to which most of the nrfA transcripts were assigned) taxonomy was inferred to family level, before S-utilization capacity was assigned.

## Supplementary information

Supplementary Information

Supplementary Tables 4, 5

## Data Availability

The nine metatranscriptomes described in the manuscript available on NCBI under BioProject ID PRJNA924993.
