# Supplementary material for: Simultaneous sulfate and nitrate reduction in coastal sediments
Source: ISME Commun. 2023 Mar 8;3:17. doi: 10.1038/s43705-023-00222-y (PMC9992702; doi:10.1038/s43705-023-00222-y)
Supplement: Supplementary file 1 — Supplementary Information [file 43705_2023_222_MOESM1_ESM.docx]

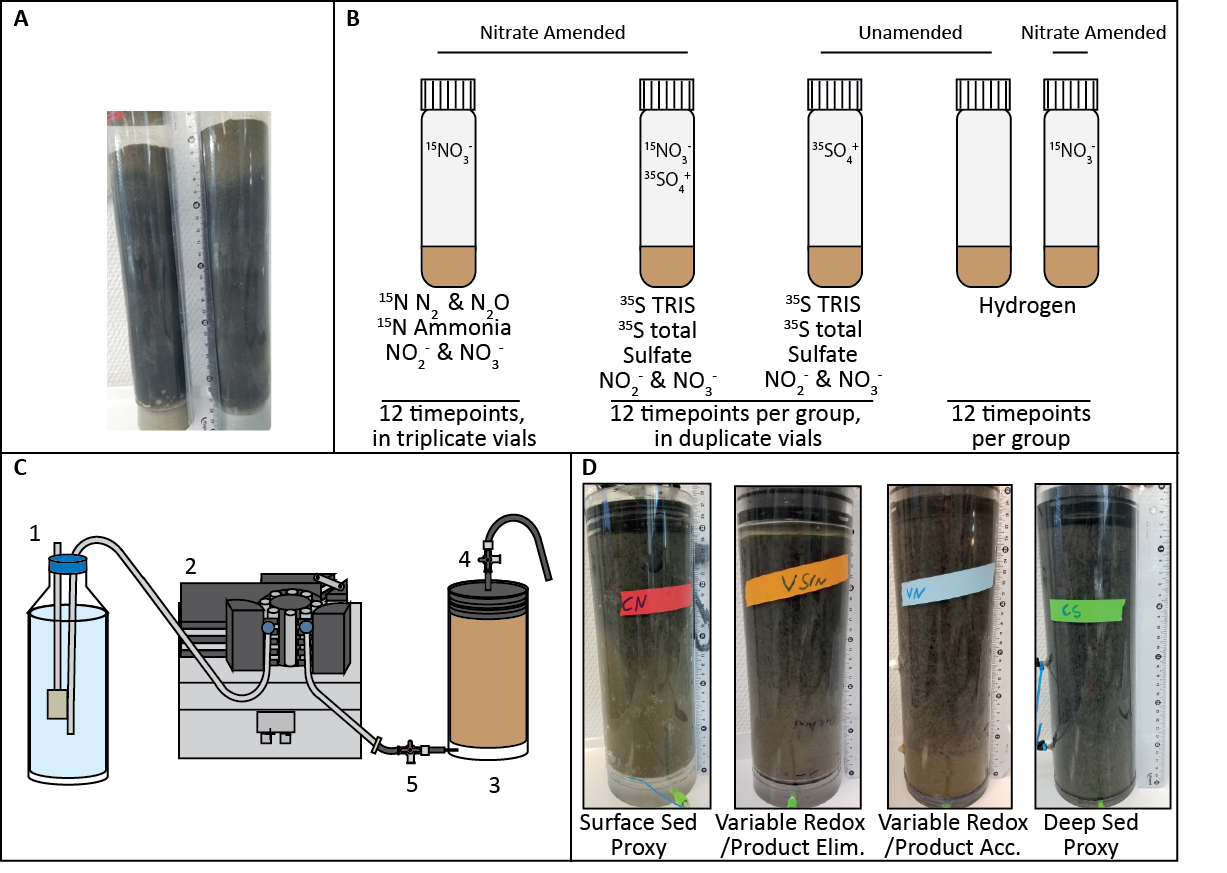


Nitrate Replete

Nitrate Deplete

Variable Redox

Product Acc.

Variable Redox

Product Elim.

**Supplemental Figure 1, methods outline:** **A.** Fresh sediment cores show an upper oxidized (pale) layer and deeper reduced (dark) layer. **B.** The different sediment incubations, and the compounds measured from each incubation. **C.** A schematic for the flow through core. Deoxygenated seawater (1) is pumped via a peristaltic pump, which is on a timer,(2) into the core base (3) which has radial grooving and a nylon grid sheet to improve plug flow. Water exits via the top of the core (4). In the variable redox / Product Elim. core, a second seawater water source, containing no nitrate, was connected to the core via the stopcock (5) and passed through a different peristaltic pump, on a different timer. **D.** The four conditioned cores before beginning the incubation experiments showed visual differences, despite an identical starting appearance.


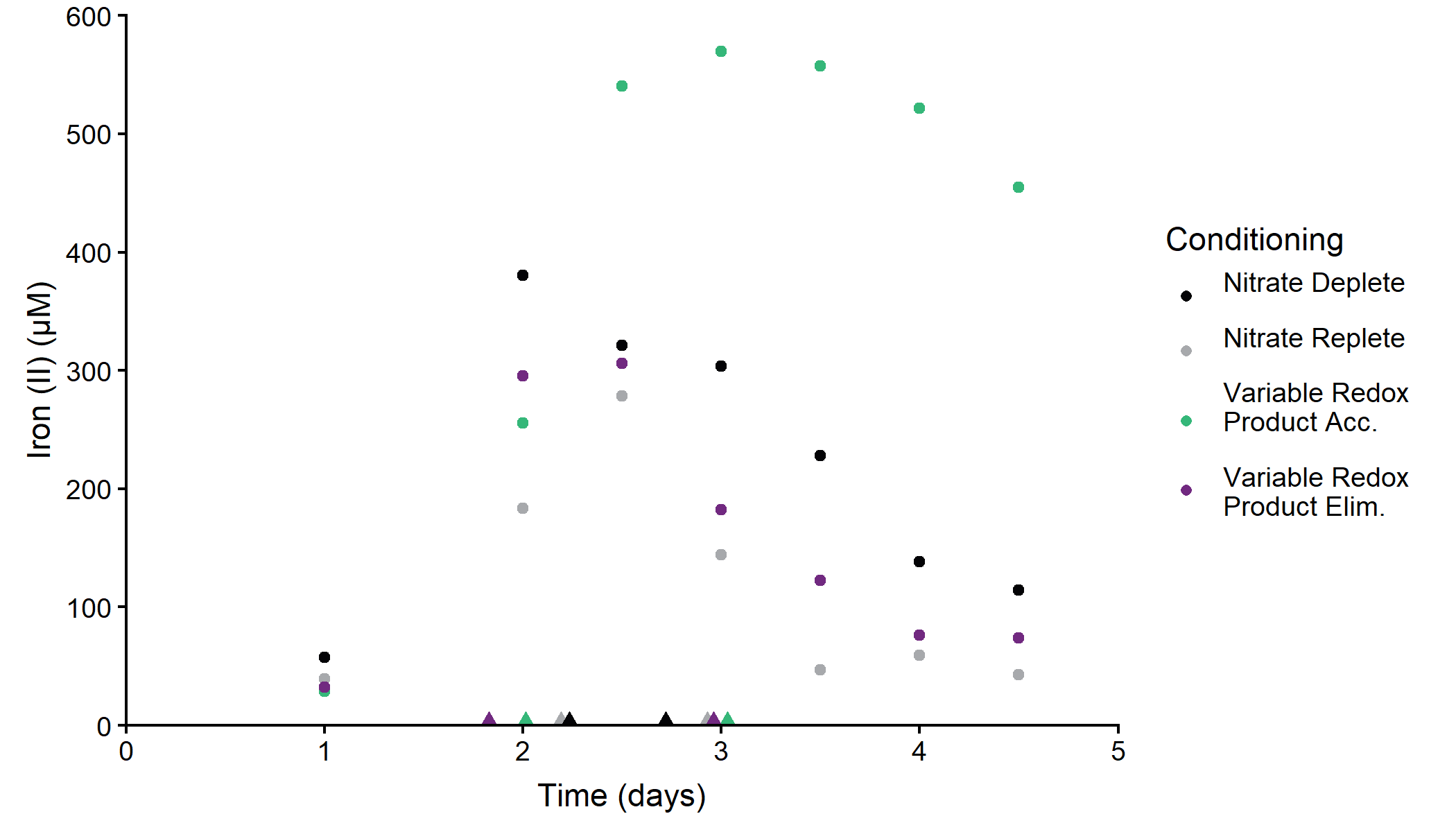


**Supplemental Figure 2, Fe II output from cores:** Porewater was collected anoxically from the cores during conditioning and reacted with ferrozine to determine iron II concentrations. Iron II concentrations over the conditioning period is plotted in micromoles/liter. Note that the nitrate concentrations in the nitrate amended cores was increased in the second day of conditioning. The triangles (bottom) represent inlet water, and the circles outlet water. The inlet water points are jittered for legibility. The deep sediment proxy iron is plotted in black, the surface sediment proxy in silver, the variable redox / Product Acc. in green, the variable redox / Product Elim. in purple.


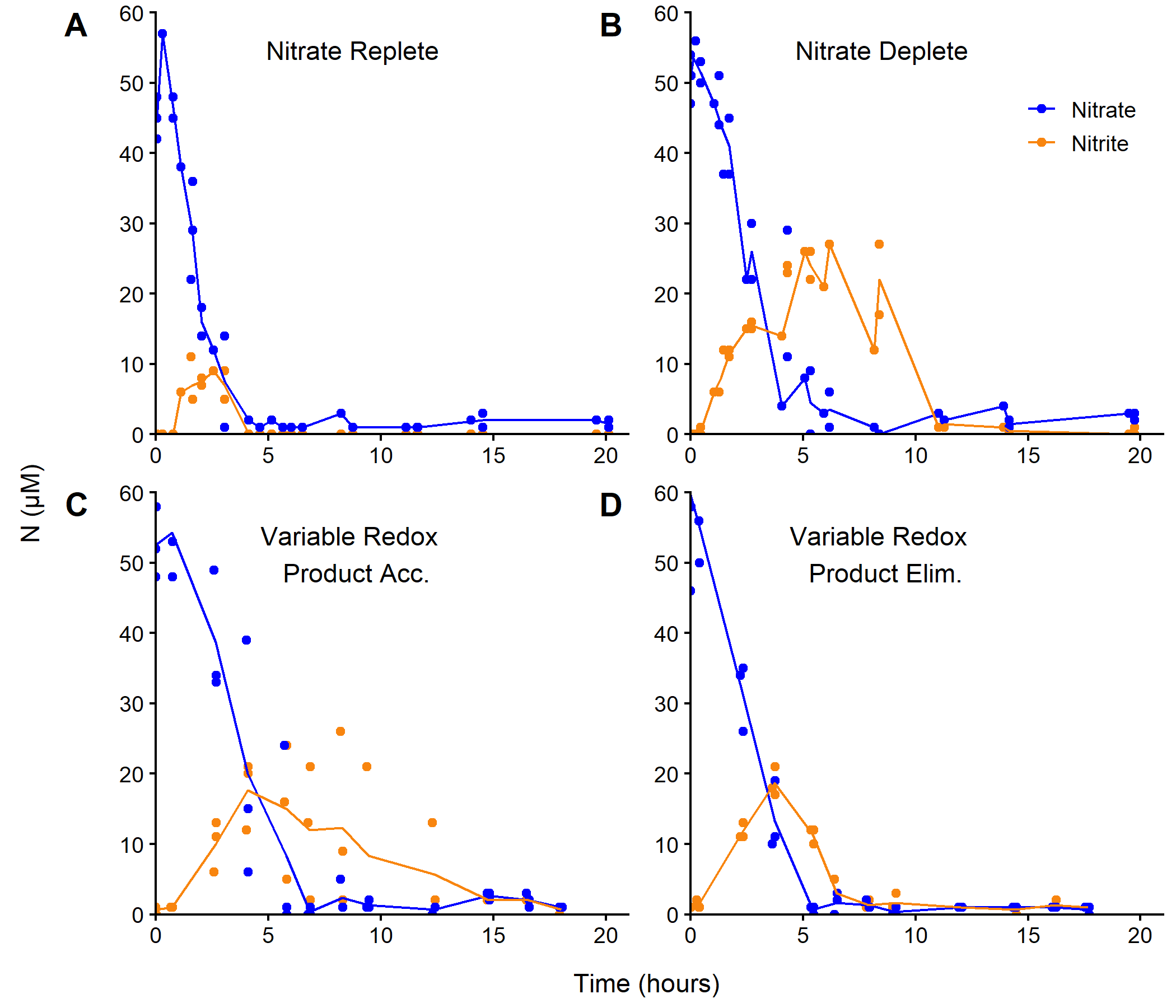


**Supplemental Figure 3, Nitrate and nitrite dynamics in conditioned sediment:** The concentration of nitrate (blue) and nitrite (orange) in the ^15^N nitrate amended samples is plotted over the incubation time. There is a clear ntrite peak in all incubations. Each point corresponds to a different Exetainer. Lines connect the median value of triplicate Exetainers.


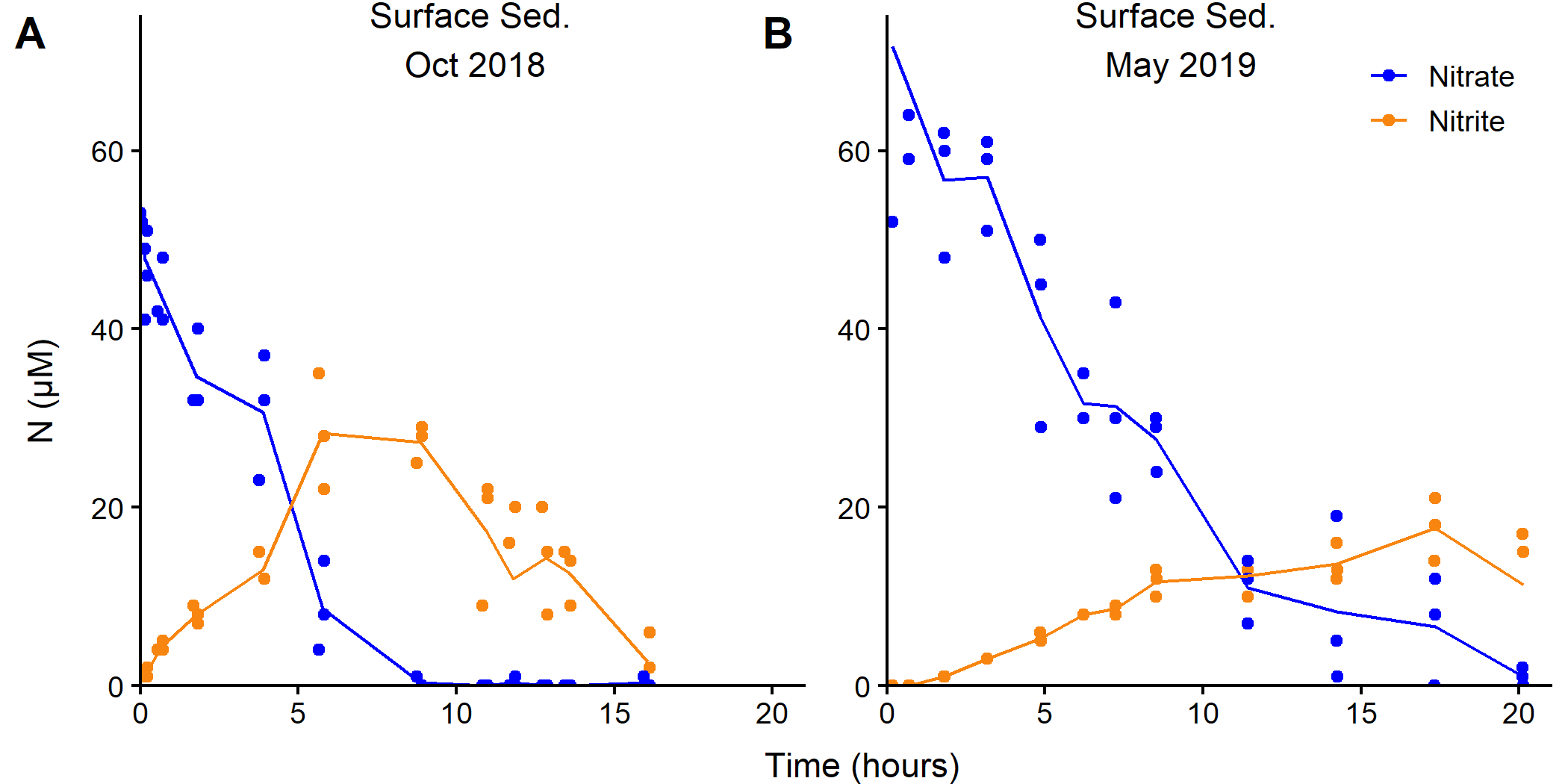


**Supplemental Figure 4, Nitrate and nitrite concentrations in fresh sediment incubations:** The concentration of nitrate (blue) and nitrite (orange) in the 15N nitrate amended samples is plotted over the incubation time. Each point corresponds to a different Exetainer, lines connect the median value of triplicate Exetainers.


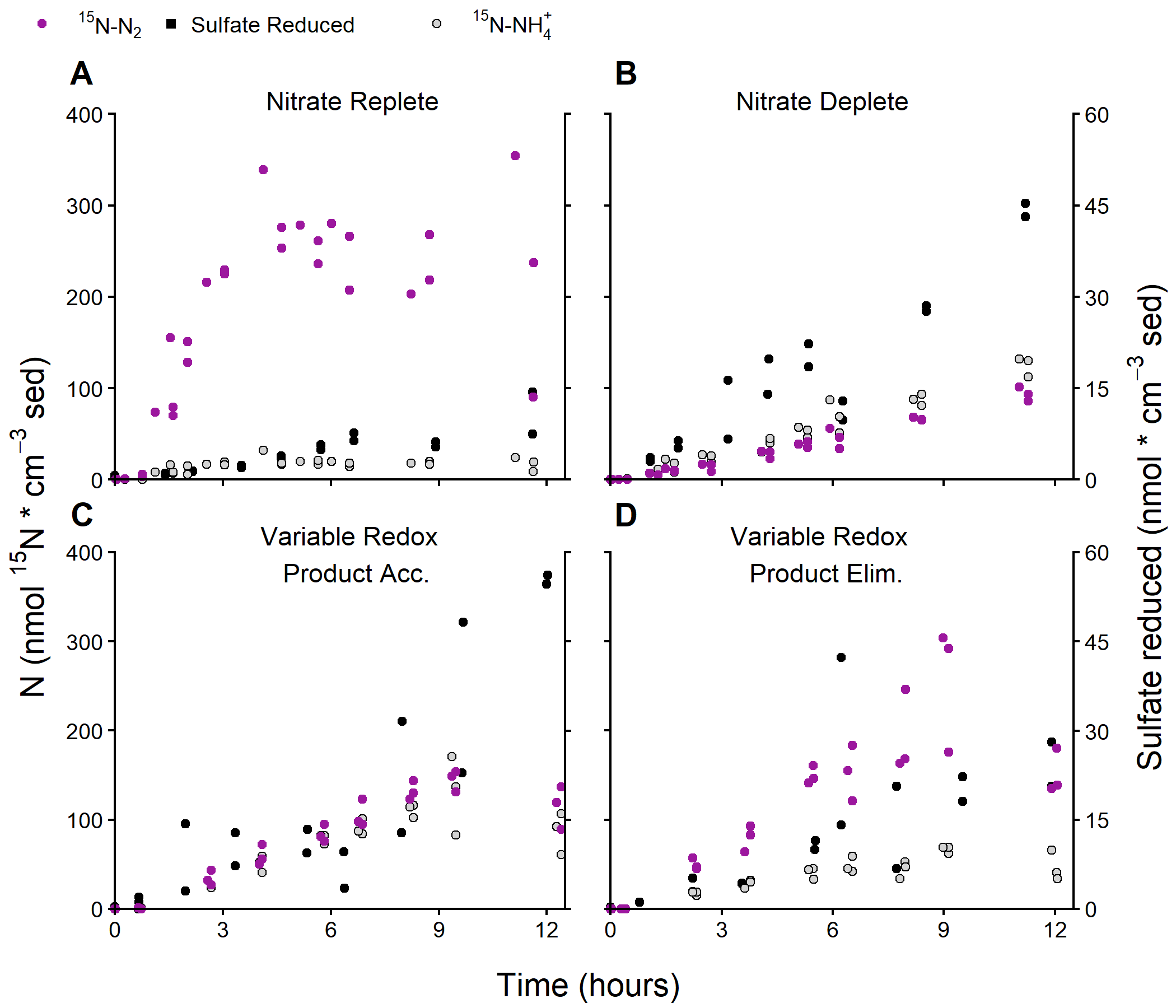


**Supplemental Figure 5, Sulfate reduction and reduced N production in conditioned sediments:** ^15^N_2_ (light grey), ^15^NH_4_^+^ (purple), and the total sulfate reduced (black) are plotted for parallel nitrate amended incubations of surface sediment collected in May 2019. Each point represents a measurement from a separate incubation.


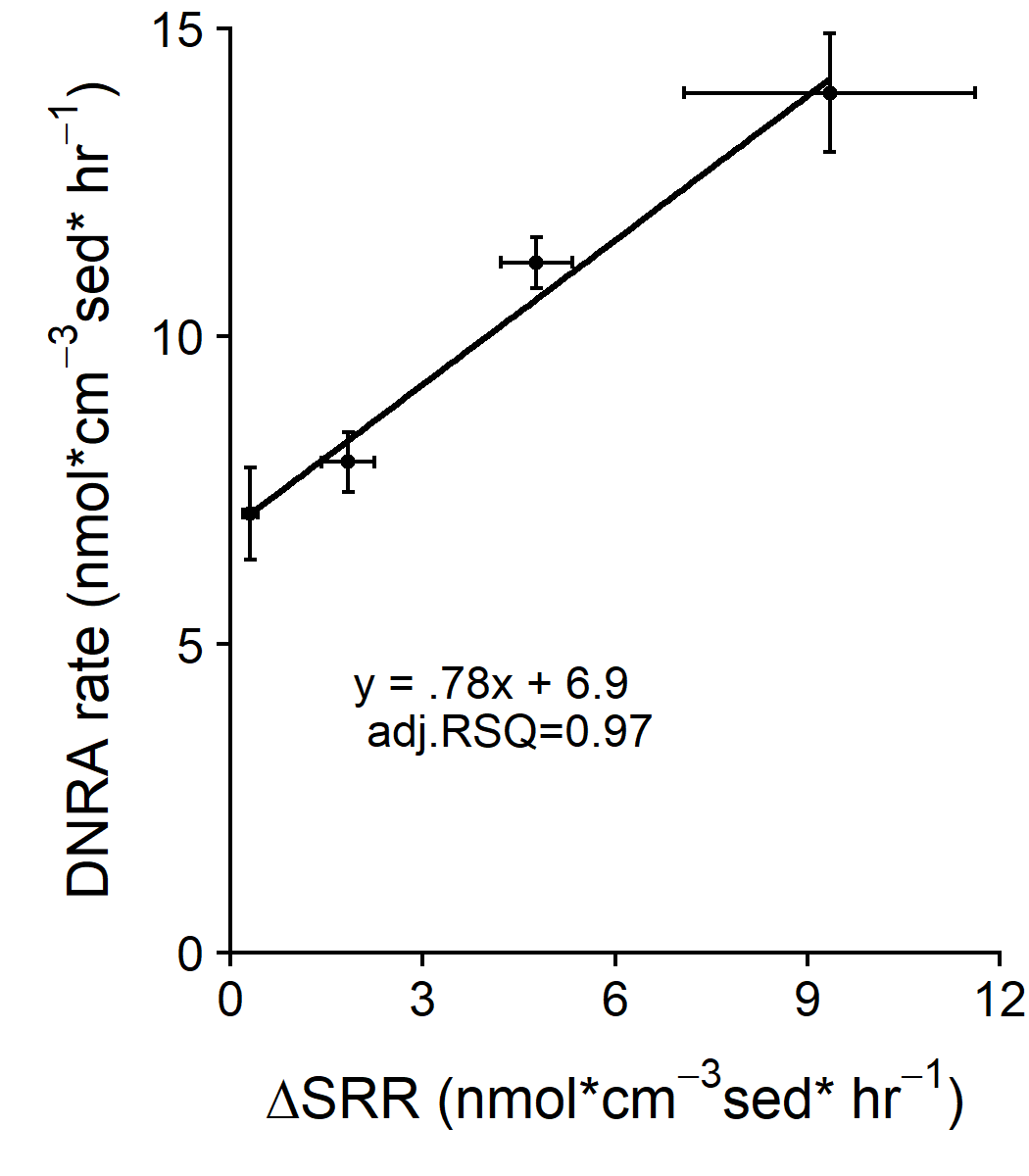


**Supplemental Figure 6, Sulfate reduction and DNRA:** The difference in sulfate reduction rates in the NO_3_^-^ amended incubations and the parallel NO_3_^-^-free incubations plotted against the rate of ^15^N ammonia production. All rates fit with an R^2^ of at least 0.85, except for the Variable Redox + Product Build Up condition, which had an R^2^ of 0.56 ad 0.75 for sulfate reduction in the presence and absence of NO_3_^-^, respectively. Vertical bars represent standard error and horizontal bars are the standard errors of sulfate reduction rates added in quadrature.


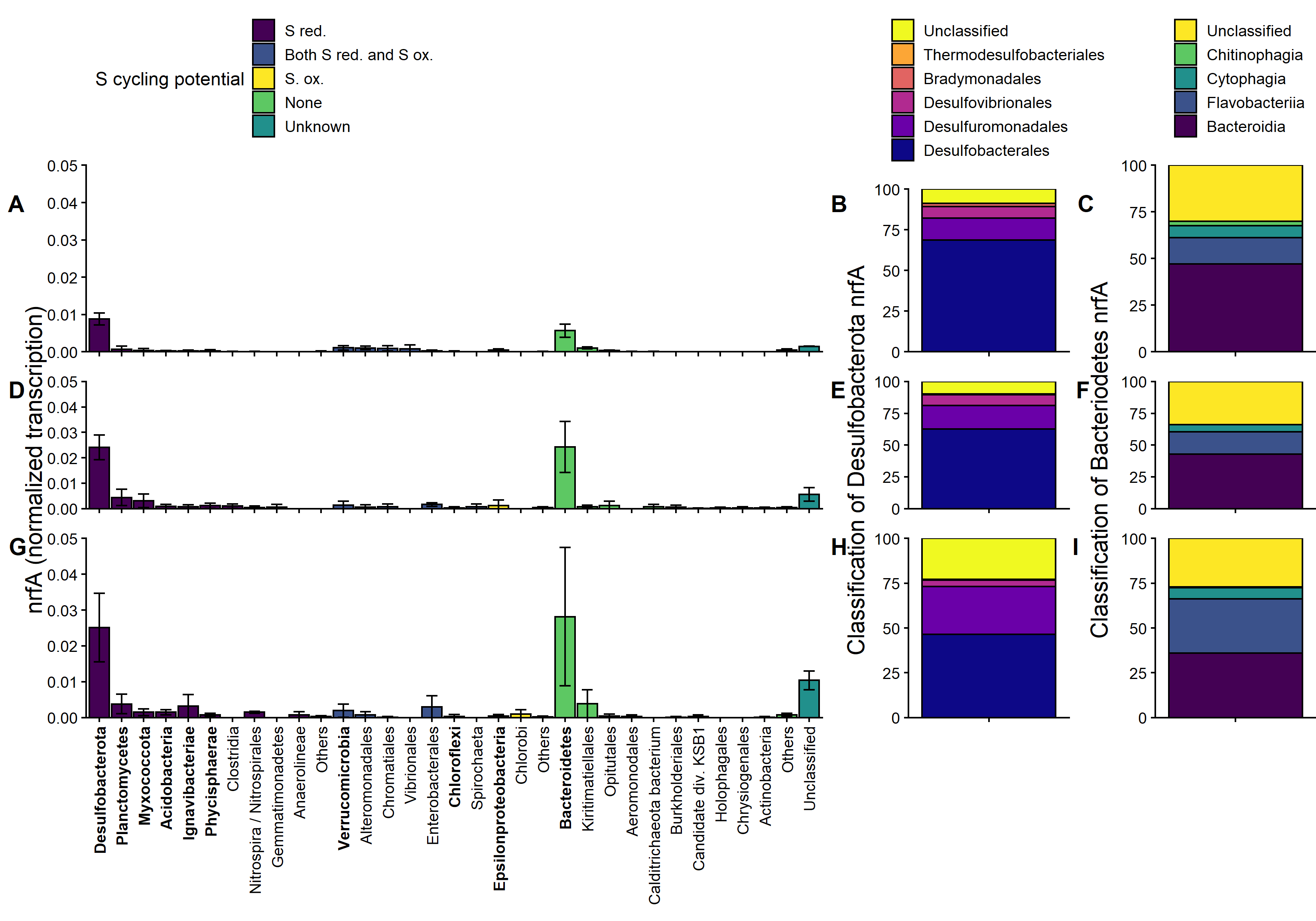


**Supplementary Figure 7:** a) Assignment of nrfA transcripts to phylum (bold), or order level. The colours indicate the potential of these classes to carry out sulfur metabolism as identified from literature surveys. Transcript abundance was normalized by gene length and against the total abundance of rpoB in the metatranscriptome. B) Assignment of Desulfobacterota nrfA transcripts to order level, as a percentage of total nrfA transcripts assigned to Desulfobacterota. C) Assignment of Bacteriodetes nrfA transcripts to order level, as a percentage of total nrfA transcripts assigned to Bacteriodetes. In both panels averages are shown from three individual metatranscriptomes and error bars are standard deviation. The top row (A-C) corresponds to the upper layer of sediment (0-1 cm depth), the middle row (D-F) to the middle layer of sediment (2-4 cm depth), and the bottom layer of sediment (6-8 cm depth).


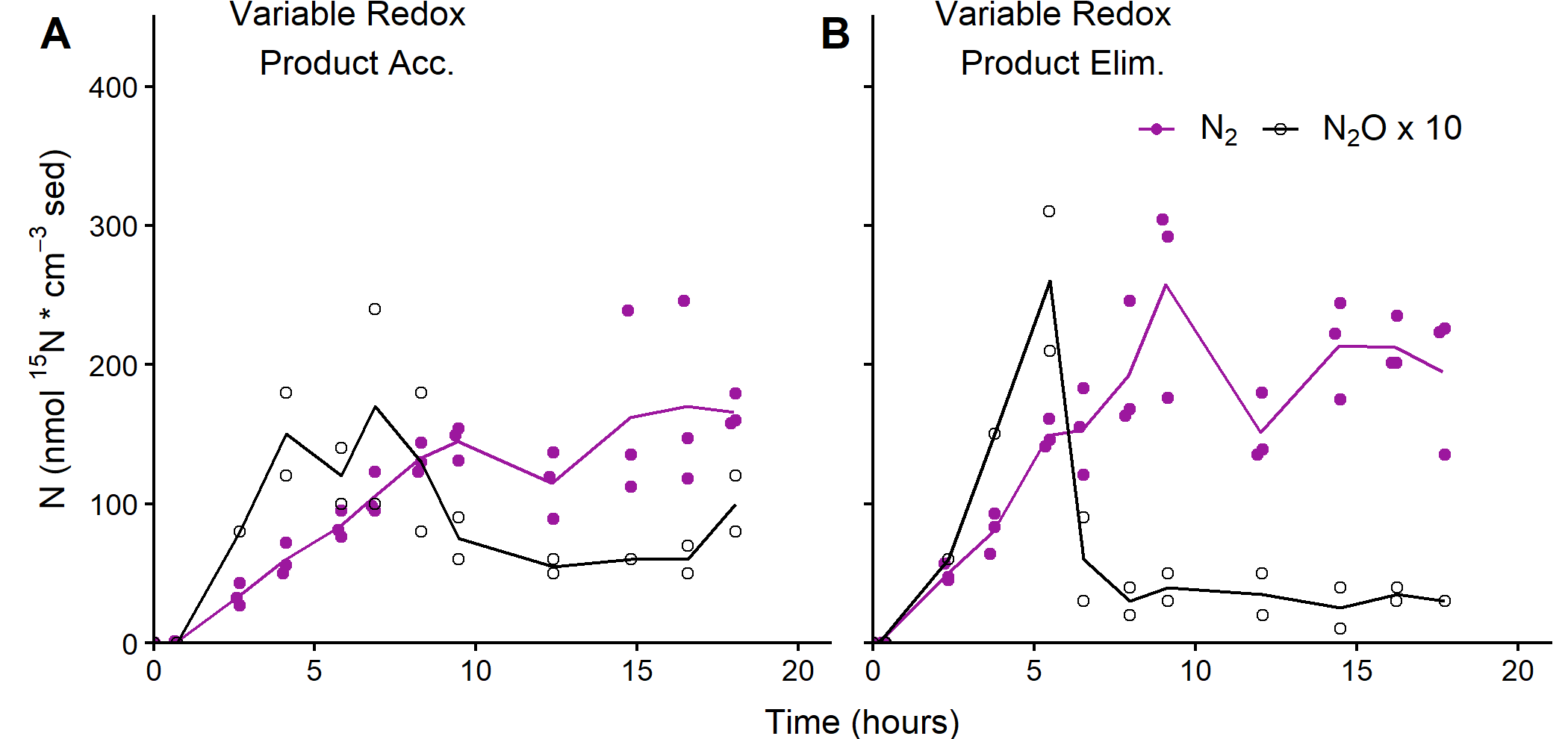


**Supplemental Figure 8, N_2_ and N_2_O production in conditioned sediments:** The production of N_2_ (purple) and N_2_O (orange, values multiplied by 10) sediments is plotted over the entire incubation time in nmol ^15^N cm-^3^ sediment. Lines connect the average value at each timepoint. Each point represents a measurement from a separate incubation.


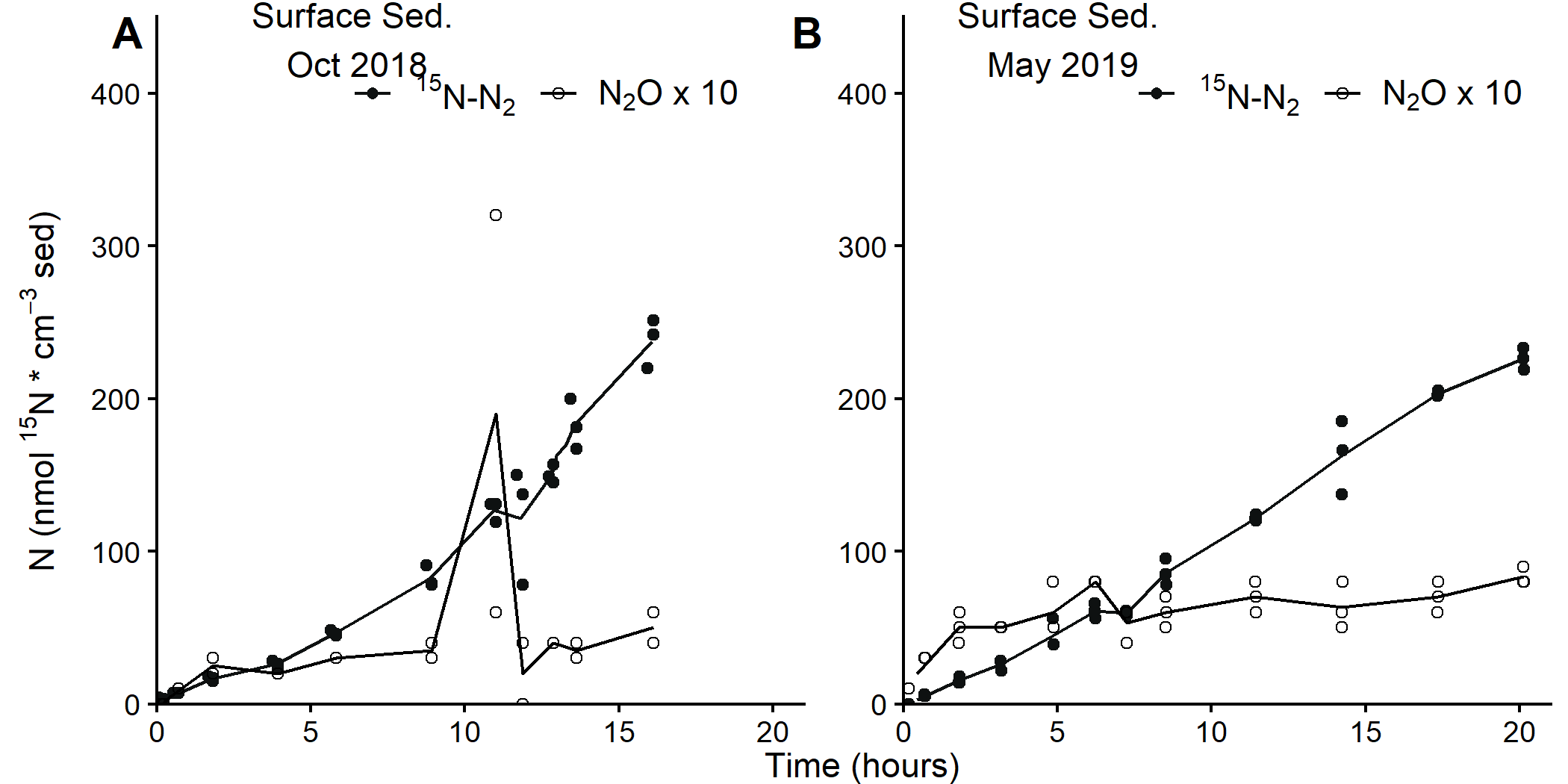


**Supplemental Figure 9, N_2_ and N_2_O production in fresh sediments:** The production of N_2_ (Solid circles) and N_2_O (open circles, values multiplied by 10) sediments is plotted over the entire incubation time in nmol ^15^N cm-^3^ sediment. Lines connect the average value at each timepoint. Each point represents a measurement from a separate incubation. Each point represents a measurement from a separate Exetainer. Lines connect the median of replicate exetainers.

| Rate Type | Conditioning | Slope | Std Error Slope | T Value Slope | P Value Slope | Adjusted R Squared | Sigma (Residual Standard Error) | Degrees of Freedom | Intercept | Intercept Std Error | Intercept T value | Intercept P Value |
| --- | --- | --- | --- | --- | --- | --- | --- | --- | --- | --- | --- | --- |
| SR w NO_3_^-^ | Nitrate Replete | 0.54 | 0.04 | 12.48 | 1.62E-05 | 0.96 | 0.13 | 6 | 0.5 | 0.1 | 7.5 | 0.0 |
| SR w NO_3_^-^ | Nitrate Deplete | 3.64 | 0.40 | 9.16 | 2.75E-07 | 0.85 | 5.13 | 14 | -0.3 | 2.3 | -0.1 | 0.9 |
| SR w NO_3_^-^ | Variable Redox Product Acc. | 2.13 | 0.60 | 3.56 | 0.007442 | 0.56 | 3.78 | 8 | 0.6 | 2.0 | 0.3 | 0.8 |
| SR w NO_3_^-^ | Variable Redox Product Elim. | 1.83 | 0.21 | 8.68 | 2.41E-05 | 0.89 | 1.30 | 8 | 0.0 | 0.6 | 0.0 | 1.0 |
| SR no NO_3_^-^ | Nitrate Replete | 0.85 | 0.11 | 7.74 | 0.000244 | 0.89 | 0.13 | 6 | 0.6 | 0.2 | 3.5 | 0.0 |
| SR no NO_3_^-^ | Nitrate Deplete | 8.41 | 0.39 | 21.46 | 4.13E-12 | 0.97 | 5.13 | 14 | 1.2 | 1.8 | 0.6 | 0.5 |
| SR no NO_3_^-^ | Variable Redox Product Acc. | 11.47 | 2.18 | 5.26 | 0.000769 | 0.75 | 3.78 | 8 | -7.8 | 6.7 | -1.2 | 0.3 |
| SR no NO_3_^-^ | Variable Redox Product Elim. | 3.66 | 0.35 | 10.40 | 4.63E-05 | 0.94 | 1.30 | 6 | 0.0 | 0.7 | 0.0 | 1.0 |
| N_2_ | Nitrate Replete | 84.02 | 5.77 | 14.56 | 7.55E-10 | 0.93 | 27.34 | 14 | -25.0 | 11.2 | -2.2 | 0.0 |
| N_2_ | Nitrate Deplete | 8.42 | 0.28 | 30.04 | 7.59E-23 | 0.97 | 5.34 | 28 | -4.3 | 1.5 | -2.9 | 0.0 |
| N_2_ | Variable Redox Product Acc. | 15.23 | 1.06 | 14.38 | 1.77E-08 | 0.94 | 7.08 | 11 | -5.0 | 3.0 | -1.6 | 0.1 |
| N_2_ | Variable Redox Product Elim. | 22.61 | 1.44 | 15.67 | 2.29E-08 | 0.96 | 7.53 | 10 | -3.6 | 3.2 | -1.1 | 0.3 |
| NH_4_^+^ | Nitrate Replete | 7.12 | 0.74 | 9.61 | 1.52E-07 | 0.86 | 3.51 | 14 | -1.9 | 1.4 | -1.3 | 0.2 |
| NH_4_^+^ | Nitrate Deplete | 11.20 | 0.42 | 26.87 | 1.56E-21 | 0.96 | 7.94 | 28 | -3.7 | 2.2 | -1.7 | 0.1 |
| NH_4_^+^ | Variable Redox Product Acc. | 13.95 | 0.96 | 14.52 | 1.61E-08 | 0.95 | 6.42 | 11 | -5.6 | 2.7 | -2.0 | 0.1 |
| NH_4_^+^ | Variable Redox Product Elim. | 7.96 | 0.48 | 16.45 | 1.44E-08 | 0.96 | 2.53 | 10 | -1.1 | 1.1 | -1.0 | 0.3 |

**Supplemental Table 1 (above), Conditioned sediment rate statistics:** The statistics for the linear regressions fitted to sulfate reduction with (SR w NO_3_^-^) and without (SR no NO_3_^-^) added nitrate, N_2_ production, and NH_4_^+^ production . Rates were calculated for the period of time where both sulfate reduction and N2 production were linear, the cutoff and start times are in Sup. Table 3. N_2_ and NH_4_^+^ production represent only the production from ^15^N labeled NO_3_^-^ while sulfate reduction represents total net sulfate reduction, determined by ^35^S sulfate reduction.

| Rate Type | Conditioning | Slope | Std Error Slope | T Value Slope | P Value Slope | Adjusted R Squared | Sigma (Residual Standard Error) | Degrees of Freedom | Intercept | Intercept Std Error | Intercept T value | Intercept P Value |
| --- | --- | --- | --- | --- | --- | --- | --- | --- | --- | --- | --- | --- |
| SR w NO_3_^-^ | May 2019 Surface Sed | 0.32 | 0.02 | 14.17 | 1.54E-12 | 0.90 | 0.68 | 22 | 0.74 | 0.23 | 3.28 | 0.00 |
| SR w NO_3_^-^ | Oct 2018 Surface Sed | 0.98 | 0.09 | 11.35 | 0.000343 | 0.96 | 1.05 | 4 | 0.05 | 0.86 | 0.05 | 0.96 |
| SR no NO_3_^-^ | May 2019 Surface Sed | 1.64 | 0.12 | 13.66 | 3.19E-12 | 0.89 | 0.68 | 22 | -0.71 | 1.22 | -0.58 | 0.57 |
| SR no NO_3_^-^ | Oct 2018 Surface Sed | 3.05 | 0.27 | 11.15 | 5.83E-07 | 0.92 | 1.05 | 10 | -3.68 | 2.90 | -1.27 | 0.23 |
| N_2_ | May 2019 Surface Sed | 11.75 | 0.26 | 44.90 | 7.44E-32 | 0.98 | 9.87 | 34 | -9.54 | 2.66 | -3.59 | 0.00 |
| N_2_ | Oct 2018 Surface Sed | 13.29 | 0.64 | 20.82 | 6.24E-21 | 0.93 | 21.36 | 34 | -12.18 | 5.81 | -2.10 | 0.04 |
| NH_4_^+^ | May 2019 Surface Sed | 4.99 | 0.24 | 20.68 | 7.76E-21 | 0.92 | 9.11 | 34 | -0.01 | 2.46 | 0.00 | 1.00 |
| NH_4_^+^ | Oct 2018 Surface Sed | 6.12 | 0.27 | 22.33 | 6.78E-22 | 0.93 | 9.17 | 34 | -2.79 | 2.50 | -1.12 | 0.27 |

**Table 2, Fresh sediment rate statistics:** The statistics for the linear regressions fitted to sulfate reduction with and without added nitrate, N2 production, and NH4 production. Rates were calculated for the period of time where both sulfate reduction and N2 production were linear, the cutoff and start times are in Sup. Table 3. N_2_ and NH_4_^+^ production represent only the production from ^15^N labeled NO_3_^-^ while sulfate reduction represents total net sulfate reduction, determined by ^35^S sulfate reduction.

| Conditions | Nitrate Replete | Nitrate Deplete | Variable Redox Product Acc. | Variable Redox Product Elim. | Oct 2018 Surface Sed | May 2019 Surface Sed |
| --- | --- | --- | --- | --- | --- | --- |
| Cut off time for rate calculations | 4.13 | 11.28 | 5.73 | 5.33 | 16.12 | 20.13 |

**Table 3 Time cutoffs for rate calculations:** Rates were calculated for the portion of the incubation where both N2 production and sulfate reduction were approximately linear. The cutoff time, after which points were not included in rate calculations, are listed for each sediment condition. The number of samples used to determine each rate depends on the sediment and the rate (N or S) and is equal to the column “degrees of freedom” + 2 in tables 1 & 2.

| Conditioning | Mean N_2_O at last timepoint (nmol ^15^N N_2_O/cm^3^ sed) | Percentage of mostly reduced nitrate in N_2_O | Percentage of nitrate comitted to DN in N_2_O |
| --- | --- | --- | --- |
| Nitrate Replete | 1 | 0.4 | 0.4 |
| Nitrate Deplete | 9.5 | 4.5 | 10.2 |
| Variable Redox Product Acc. | 10 | 3.6 | 5.6 |
| Variable Redox Product Elim. | 3 | 1.3 | 1.6 |
| Oct 2018 Surface Sed | 5 | 1.4 | 2.0 |
| May 2019 Surface Sed | 8.3 | 2.6 | 3.6 |

**Supplementary Table 6, N_2_O remaining at the end of incubations:** The average remaining ^15^N N_2_O in nmol ^15^N N_2_O/cm^3^ sediment at the end of incubations, the percentage of mostly reduced nitrate (N_2_ +N_2_O +NH_4_^+^) in N_2_O at the end of incubations, and the percentage of nitrate committed to denitrification (N_2_ + N_2_O) remaining in N_2_O is displayed.

|  | Complete nitrate reduction rate | Flow rate | 0-4 cm volume | Nitrate cleared per 0-4 cm | Concentration nitrate cleared 0-4 cm | 4-10 cm volume | Nitrate cleared 4-10 cm | Concentration nitrate cleared 4-10 cm | Exposure at 4 cm with 200 µM amendment | Exposure at 4 cm with 400 µM amendment | Exposure at 10 cm with 200 µM | Exposure at 10 cm with 400 µM |
| --- | --- | --- | --- | --- | --- | --- | --- | --- | --- | --- | --- | --- |
| Unit | (nmol 15N NH4 + N2 produced /cm3*sed) | mL/  hr | cm3 sed | µmol/hr | µM/hr | cm3 sed | µmol/hr | µM/hr | µM/hr | µM/hr | µM/hr | µM/hr |
| Oct 2018 Surface Sed | 19 | 48 | 254 | 5 | 101 | 382 | 7 | 151 | 99 | 299 | 49 | 249 |
| Nitrate Replete | 91 | 47 | 254 | 23 | 493 | 382 | 35 | 739 | -293 | -93 | -539 | -339 |
| Nitrate Deplete | 20 | 48 | 254 | 5 | 106 | 382 | 8 | 159 | N/A | N/A | N/A | N/A |
| Variable Redox Product Acc. | 29 | 48 | 254 | 7 | 154 | 382 | 11 | 231 | 46 | 246 | -31 | 169 |
| Variable Redox Product Elim. | 31 | 48 | 254 | 8 | 164 | 382 | 12 | 247 | 36 | 236 | -47 | 153 |

**Supplementary Table 7, Minimum nitrate exposure during conditioning:** Using the rate of complete nitrate reduction to N_2_ and NH_4_^+^, the minimum nitrate exposure of the tested zone in the conditioning cores (4-10 cm) is calculated at the bottom and top of the core. The surface sediment from October 2018 is included as a reference for the beginning of the conditioning period.
